# Supplementary material for: A proximity proteomics pipeline with improved reproducibility and throughput
Source: Mol Syst Biol. 2024 Jul 1;20(8):952–71. doi: 10.1038/s44320-024-00049-2 (PMC11297269; doi:10.1038/s44320-024-00049-2)
Supplement: Supplementary file 1 — Appendix [file 44320_2024_49_MOESM1_ESM.pdf]

A proximity proteomics pipeline with improved reproducibility and throughput

Authors

Xiaofang Zhong[1,2,3,†], Qiongyu Li[1,2,3,†], Benjamin J. Polacco[1,2,3], Trupti Patil[1,2,3], Aaron Marley[4], Helene Foussard[1,2,3], Prachi Khare[1,2,3], Rasika Vartak[1,2,3], Jiewei Xu[1,2,3], Jeffrey F. DiBerto[5], Bryan L. Roth[5], Manon Eckhardt[1,2,3], Mark Von Zastrow[1,3,4], Nevan J. Krogan[1,2,3], Ruth Hüttenhain[1,2,3,6]

Affiliations

[1]Quantitative Biosciences Institute (QBI), University of California, San Francisco, San Francisco, CA 94158, USA.
[2]J. David Gladstone Institutes, San Francisco, CA 94158, USA.
[3]Department of Cellular and Molecular Pharmacology, University of California, San Francisco, San Francisco, CA 94158, USA.
[4]Department of Psychiatry and Behavioral Sciences, University of California, San Francisco, CA 94158, USA.
[5]Department of Pharmacology, School of Medicine, University of North Carolina at Chapel Hill, Chapel Hill, NC 27599, USA.
[6]Department of Molecular and Cellular Physiology, Stanford University, Stanford, CA 94305, USA.

[†]Authors contributed equally

Correspondence

Ruth Hüttenhain (ruthh@stanford.edu)

Table of contents

Content	Title	Page
Appendix Figure S1	Schematic of compartment-specific APEX2-tagged constructs	3
Appendix Figure S2	Sample enrichment and DIA-mass spectrometry method optimization.	4
Appendix Figure S3	Comparison of manual- and automated- enrichment methods coupled to DDA- and DIA-mass spectrometry methods.	5
Appendix Figure S4	Characterization of all selected monoclonal APEX2-tagged localization domain cell lines.	6
Appendix Figure S5	APEX-labeling proteomics quality control.	7
Appendix Figure S6	Volcano plots for APEX2 proximity labeling of each subcellular compartment.	8
Appendix Figure S7	Mapping ligand-dependent proximal interaction network changes of $5HT_{2A}$.	9
Appendix Figure S8	$5HT_{2A}$ network dynamics for sustained, activity-dependent proximal interactions.	10
Appendix Figure S9	Flow cytometric analysis of $5HT_{2A}$ receptor at the plasma membrane.	11
Appendix Table S1	Performance comparison of the automated PL strategy combined with DIA-based MS.	12
Appendix Table S2	Summary of optimized conditions in high-input vs. low-input proximity proteome pipeline.	13

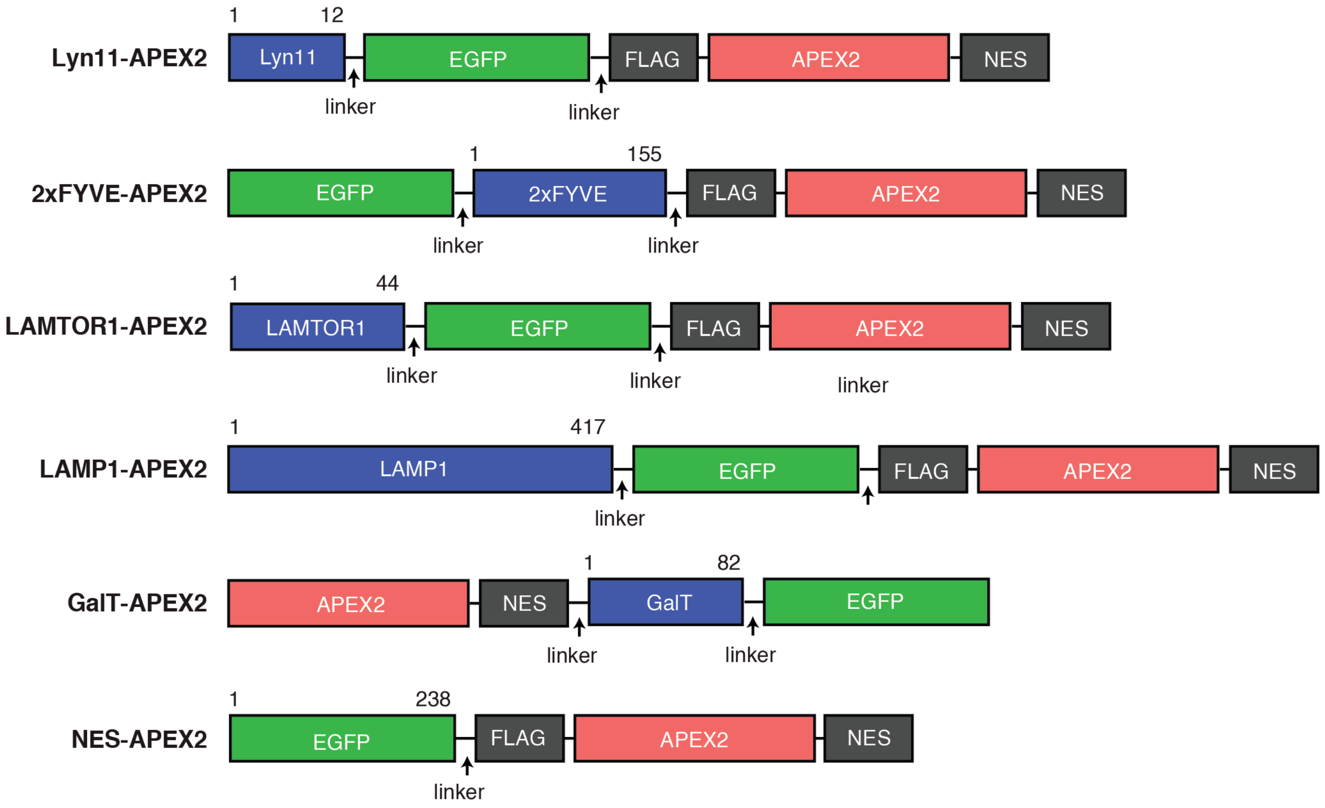

Appendix Figure S1. Schematic of compartment-specific APEX2-tagged constructs.

In our study, we selected protein localization domain Lyn11 for plasma membrane, 2xFYVE for endosome, LAMTOR1 and LAMP1 targeting sequence for late endosome/lysosome, and β-1,4 galactosyltransferase (GalT) for Golgi apparatus. A non-location specific construct for the cytosol was used as a control.

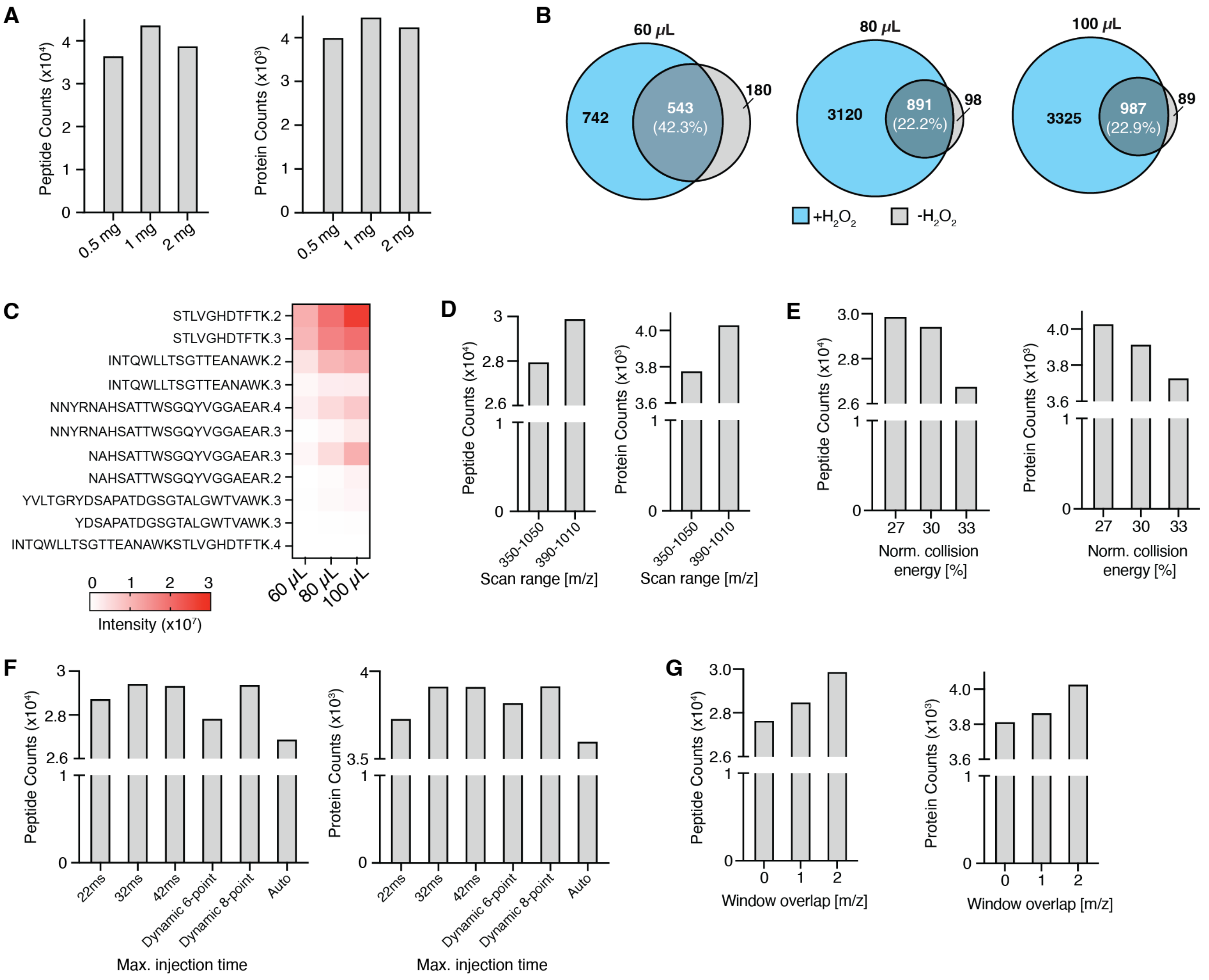

Appendix Figure S2. Sample enrichment and DIA-mass spectrometry method optimization.

A, Different protein loading amounts with 100 μL beads. **B**, Venn diagram of proteins identified with vs. without H_2O_2 treatment using different beads. **C**, Line chart of streptavidin peptides intensities. Three replicates for each condition. **D-G**, Optimization of DIA-mass spectrometry parameters including: Scan range (**D**), Normalized collision energy (**E**), Maximum injection time (**F**), Width of overlapping window (**G**).

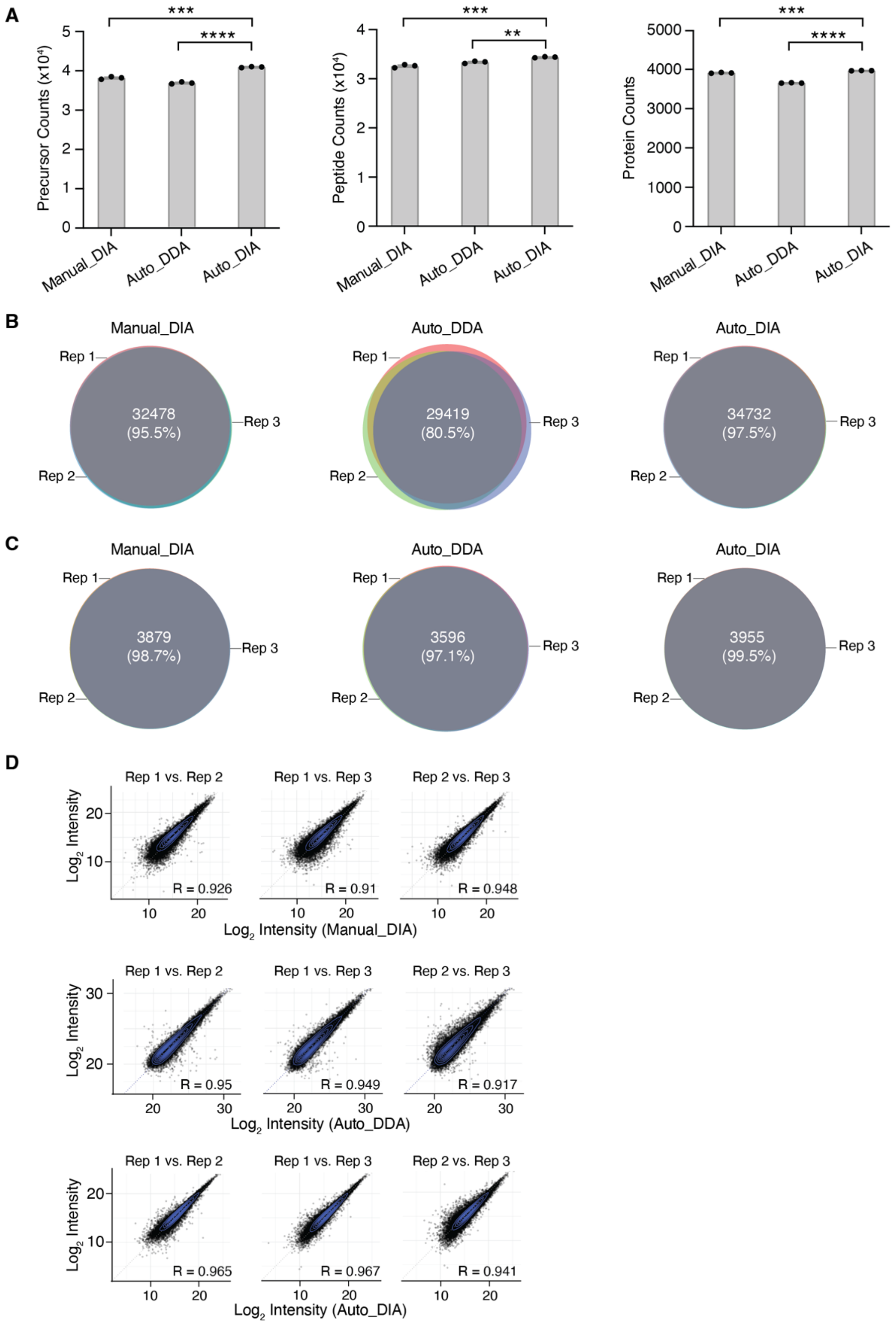

Appendix Figure S3. Comparison of manual- and automated- enrichment methods coupled to DDA- and DIA-mass spectrometry methods.

A, Number of precursors, peptides, and proteins being identified in the three methods. Statistical significance was performed in Prism (GraphPad) using unpaired t-test. **B**, Venn diagram analysis comparing peptide identification across three replicates. **C**, Venn diagram analysis comparing protein identification across three replicates. **D**, Correlation analysis of peptide intensities.

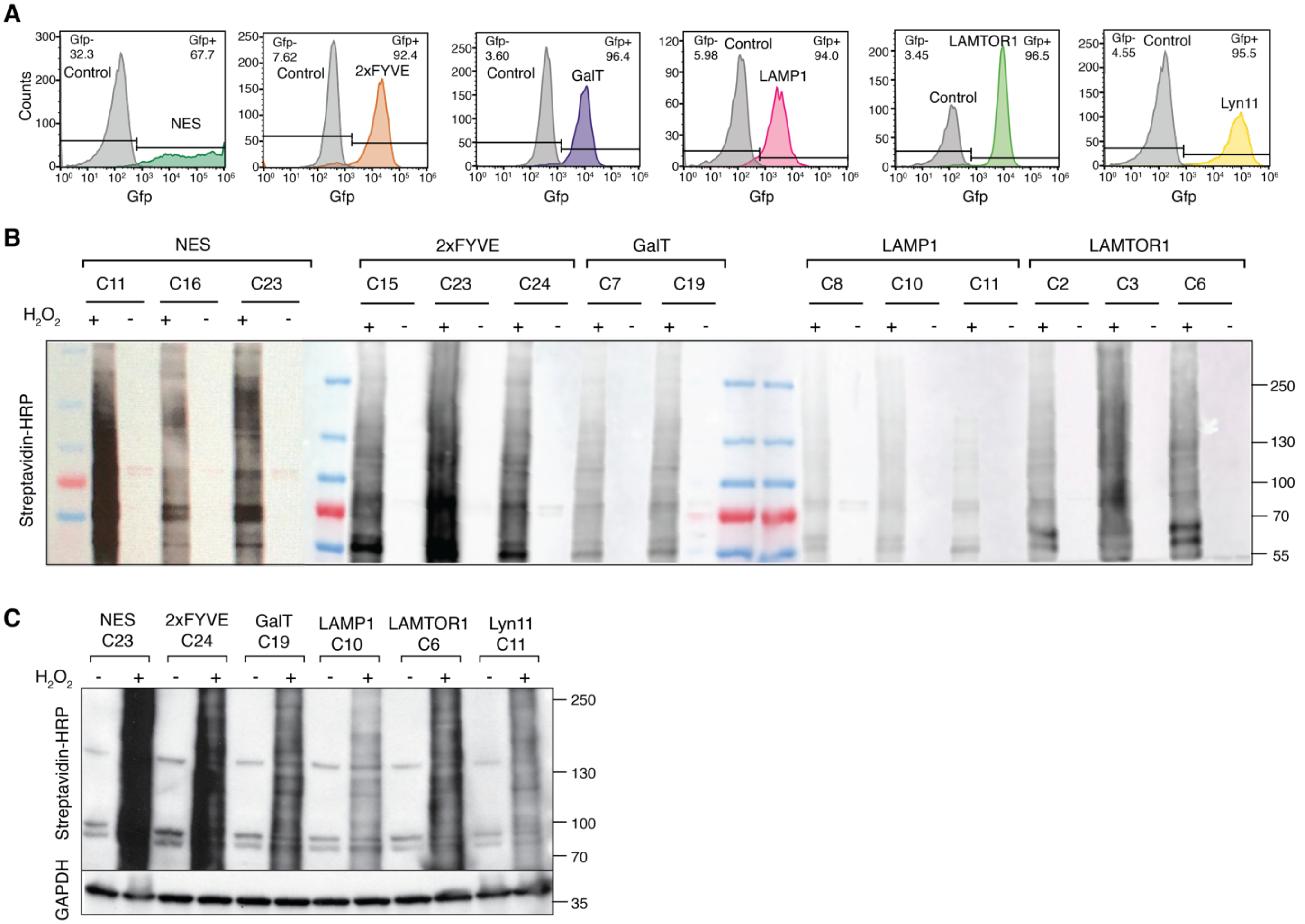

Appendix Figure S4. Characterization of all selected monoclonal APEX2-tagged localization domain cell lines.

A, Flow cytometry analysis of APEX2-tagged location domain cell lines. GFP was used to evaluate expression and localization of the APEX2 construct. The non-Doxycycline induced cell line was used as control. **B**, Western blot analysis of whole cell lysate derived from monoclonal APEX2 cell lines. **C**, Western blot analysis of final monoclonal APEX2-tagged location domain cell lines with or without H_2O_2.

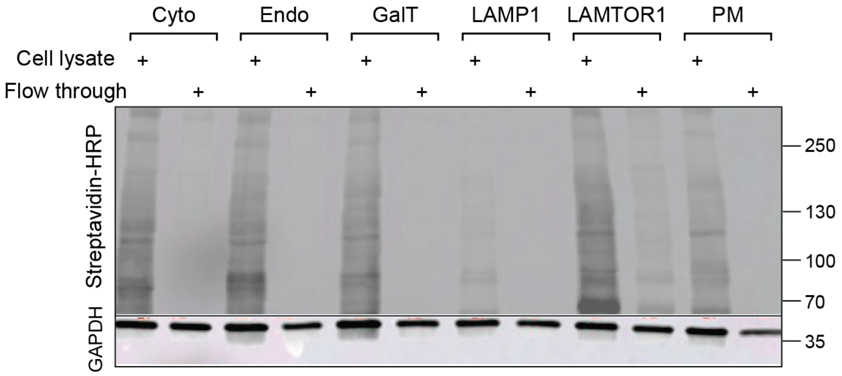

Appendix Figure S5. APEX-labeling proteomics quality control.

Western blot analysis of biotinylation and enrichment efficiency of 6 monoclonal APEX2-tagged location domain cell lines.

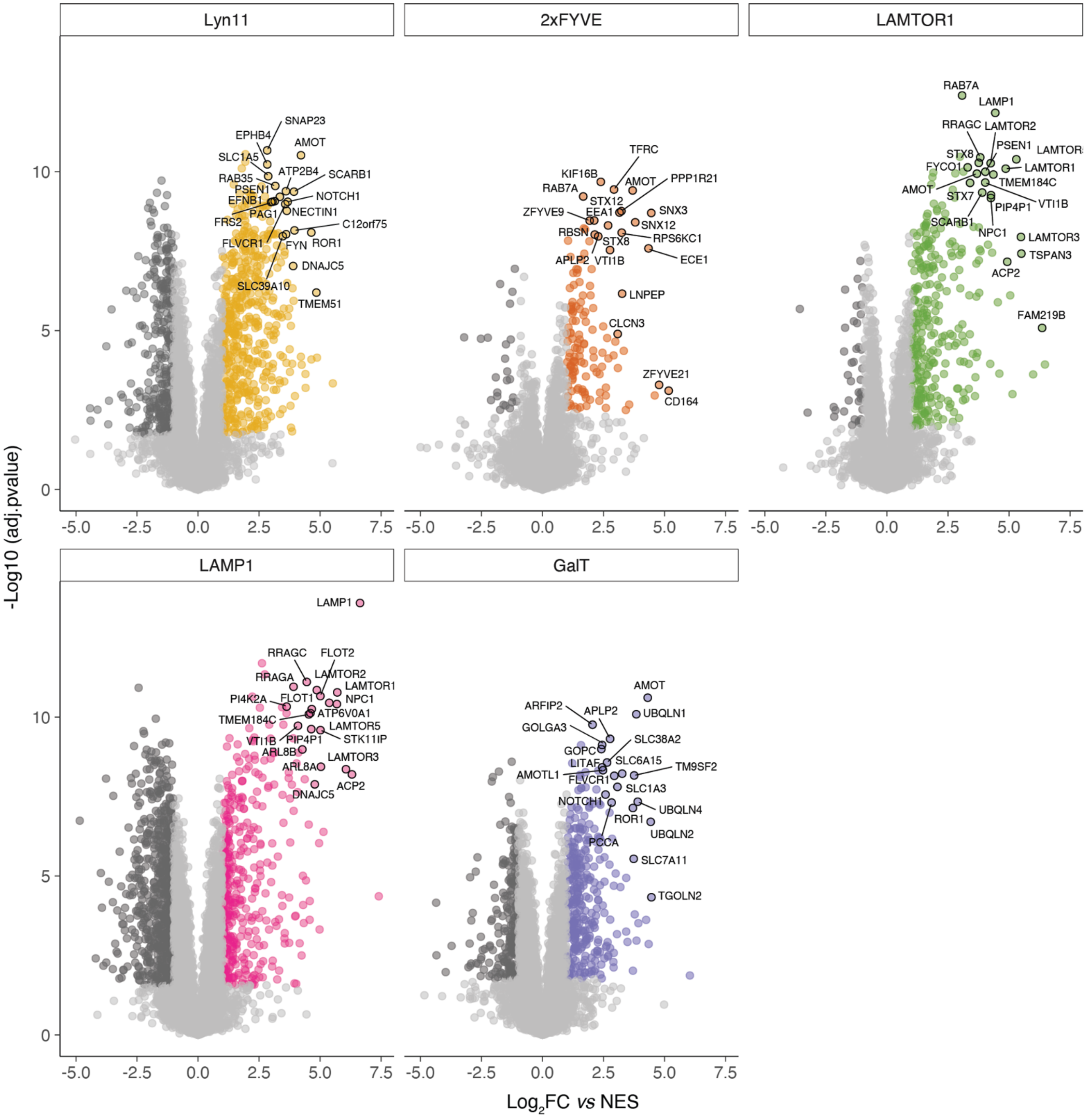

Appendix Figure S6. Volcano plots for APEX2 proximity labeling of each subcellular compartment.
Enlarged volcano plots of **Figure 4C** to annotate known location specific proteins. Proteins with a $\log_2$ fold change >1 and adjusted p-value <0.05 were considered significant and colored.

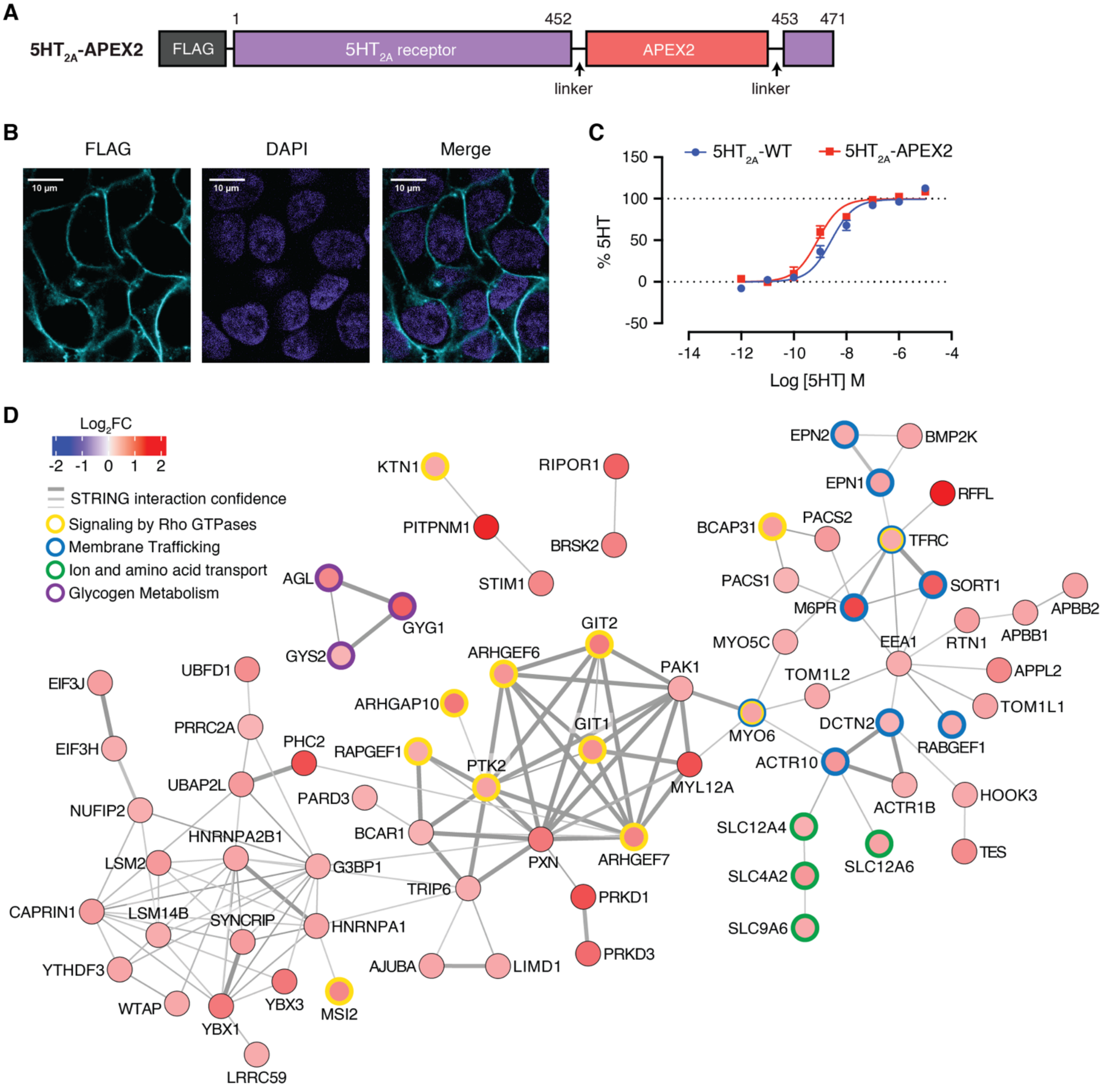

Appendix Figure S7. Mapping ligand-dependent proximal interaction network changes of 5HT$_{2A}$.

A, Construct design of 5HT$_{2A}$ receptor. **B**, Confocal imaging of monoclonal 5HT$_{2A}$-APEX2 cell lines. Cells were stained with DAPI for the nucleus and the APEX2 construct location was indicated by FLAG. Scale bar represents 10 µm. **C**, BRET validation of 5HT mediated Gq recruitment to APEX2-tagged vs. wild-type 5HT$_{2A}$. **D**, Protein interaction network connecting proteins with sustained agonist-dependent changes in the proximity of 5HT$_{2A}$. Proteins shown as nodes were colored according to their $\log_2$ fold change. The edges connecting the proteins were derived from STRING (Szklarczyk *et al*, 2011) and the edge width was scaled according to the interaction confidence. Proteins corresponding to Reactome pathways (Jassal *et al*, 2020) that were enriched within the sustained cluster were indicated with different colored node borders.

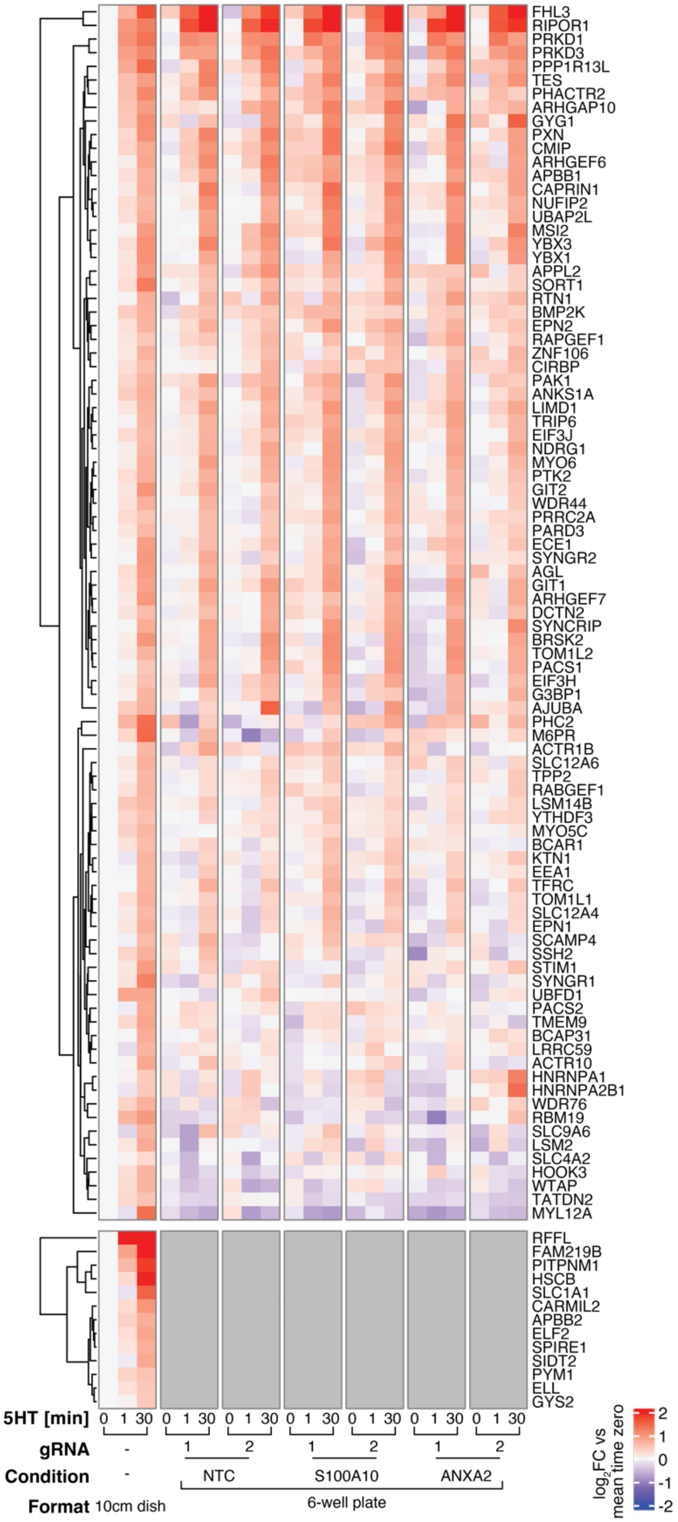

Appendix Figure S8. 5HT$_{2A}$ network dynamics for sustained, activity-dependent proximal interactions.

Heatmap depicting proteins from 5HT2A-APEX2 experiment with sustained responses to 5HT treatment (**Appendix Figure S7D**) compared across NTCs and knockouts of S100A10 and ANXA2 in 6-well plate format and the 5HT2A APEX2 data from 10cm dishes. Data were collected from three independent biological replicates (n = 3) for NTCs and S100A10 and two independent biological replicates (n = 2) for ANXA2.

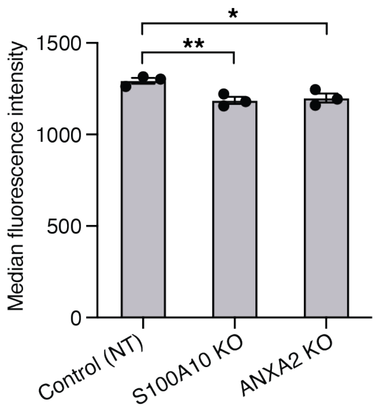

Appendix Figure S9. Flow cytometric analysis of $5HT_{2A}$ receptor at the plasma membrane.

Quantification of $5HT_{2A}$ receptor at the plasma membrane in NTC, S100A10 KO, and ANXA2 KO cells. Data from three independent experiments are presented as mean ± SEM. Statistical significance was performed in Prism (GraphPad) using unpaired t-test (NTC vs. S100A10 KO, *p* value: 0.006; NTC vs. ANXA2 KO, *p* value: 0.0161.)

	Rep 1 total	Rep 2 total	Rep 3 total	Rep 1 only	Rep 2 only	Rep 3 only	Rep 1 ∩ Rep 2	Rep 2 ∩ Rep 3	Rep 3 ∩ Rep 1	Rep 1 ∩ Rep 2 ∩ Rep 3
Manual DIA	37867	38502	38216	163	49	55	502	959	210	36992 (95%)
Auto DDA	36875	36618	36408	604	946	954	2373	1556	2155	31743 (78.7%)
Auto DIA	41058	40991	40796	45	74	55	489	217	313	40211 (97.1%)

Appendix Table S1. Performance comparison of the automated PL strategy combined with DIA-based MS.

Details for the venn diagrams of precursor features depicted in **Figure 3E**.

	High-input PL	Low-input PL
Cell culture	10cm dish	6-well plate coated with Poly-D-lysine (PDL)
Cell seeding density	4 x 10^6 cells/dish	0.5 x 10^6 cells/well
Sample input amount	1 mg	0.25 mg
Beads amount	80 µL	25 µL
Enrichment buffer volume	1 mL	200 µL
Beads washing volume	1 mL	200 µL
Protein digestion volume	200 µL	100 µL
MS sample loading amount	Resuspended in 20 µL 0.1% formic acid and inject 1 µL	Resuspended in 20 µL 0.1% formic acid and inject 3 µL

Appendix Table S2. Summary of optimized conditions in high-input vs. low-input proximity proteome pipeline. The difference between high-input and low-input proximity labeling pipeline includes reduced cell culture scale from a 10-cm dish to a 6-well plate format, decreased bead and reagent volumes throughout the enrichment and protein digestion, and increased sample injection volume for MS analysis.

References

Jassal B, Matthews L, Viteri G, Gong C, Lorente P, Fabregat A, Sidiropoulos K, Cook J, Gillespie M, Haw R, *et al* (2020) The reactome pathway knowledgebase. *Nucleic Acids Res* 48: D498–D503

Szklarczyk D, Franceschini A, Kuhn M, Simonovic M, Roth A, Minguez P, Doerks T, Stark M, Muller J, Bork P, *et al* (2011) The STRING database in 2011: functional interaction networks of proteins, globally integrated and scored. *Nucleic Acids Res* 39: D561–8
